# AP1 transcription factors are required to maintain the peripheral taste system

**DOI:** 10.1038/cddis.2016.343

**Published:** 2016-10-27

**Authors:** Jayasha Shandilya, Yankun Gao, Tapan K Nayak, Stefan G E Roberts, Kathryn F Medler

**Affiliations:** 1 Department of Biological Sciences, University at Buffalo, Buffalo, NY 14260, USA; 2 Department of Physiology & Biophysics, University at Buffalo, Buffalo, NY 14214, USA

## Abstract

The sense of taste is used by organisms to achieve the optimal nutritional requirement and avoid potentially toxic compounds. In the oral cavity, taste receptor cells are grouped together in taste buds that are present in specialized taste papillae in the tongue. Taste receptor cells are the cells that detect chemicals in potential food items and transmit that information to gustatory nerves that convey the taste information to the brain. As taste cells are in contact with the external environment, they can be damaged and are routinely replaced throughout an organism's lifetime to maintain functionality. However, this taste cell turnover loses efficiency over time resulting in a reduction in taste ability. Currently, very little is known about the mechanisms that regulate the renewal and maintenance of taste cells. We therefore performed RNA-sequencing analysis on isolated taste cells from 2 and 6-month-old mice to determine how alterations in the taste cell-transcriptome regulate taste cell maintenance and function in adults. We found that the activator protein-1 (AP1) transcription factors (c-Fos, Fosb and c-Jun) and genes associated with this pathway were significantly downregulated in taste cells by 6 months and further declined at 12 months. We generated conditional c-Fos-knockout mice to target K14-expressing cells, including differentiating taste cells. c-Fos deletion caused a severe perturbation in taste bud structure and resulted in a significant reduction in the taste bud size. c-Fos deletion also affected taste cell turnover as evident by a decrease in proliferative marker, and upregulation of the apoptotic marker cleaved-PARP. Thus, AP1 factors are important regulators of adult taste cell renewal and their downregulation negatively impacts taste maintenance.

The sense of taste is used to identify food items for consumption while avoiding potentially toxic compounds. To maintain functionality, the peripheral taste cells located in the oral cavity are continuously replaced throughout an organism's lifetime. If this renewal process is damaged, the ability to taste is impaired which negatively affects appetite and can lead to malnutrition. The efficiency of the taste cell renewal process decreases with age and can be disrupted by disease, radiation or chemotherapy which all results in taste loss or dysfunction.^1, 2, 3^ Studies using genetic lineage tracing methods have identified populations of progenitor/stem cells for taste buds, but how these cells repopulate taste buds is not well understood.^4^ Very few regulatory factors of taste cell proliferation or differentiation have been identified. Sonic hedgehog (Shh) and Wnt/β-catenin signaling pathways have been shown to have an important role in adult taste cell renewal^5, 6^ and β-catenin activity in the taste buds of 6-month-old mice was significantly lower when compared with activity levels in 10-week-old mice.^6^ Wnt/β-catenin signaling has a well-established role in cell proliferation and differentiation in embryonic taste epithelium^7^ and their role in taste cell turnover may be reminiscent of their function during development. However, the underlying mechanisms affected by Wnt signaling in adult taste cells are unclear. In general, there is very little information about the specific factors and pathways that are required to maintain adult taste cell function.

In this study, we have used an unbiased approach of sequencing messenger RNA (mRNA) isolated from taste receptor cells of the circumvallate (CV)/foliate (Fol) taste papillae to identify factors with a potential role in taste cell maintenance. We determined that the expression of the activator protein-1 (AP1) family of transcription factors (c-Fos, c-Jun and Fosb) significantly decreased in the 6 month taste cells compared with the 2 month taste cells. c-Fos couples with members of the Jun family to form AP1 transcription activator proteins which have roles in cell differentiation, proliferation and death.^8, 9, 10^ c-Fos is also a well-established early response gene that transduces short-term stimuli into long-term responses within a cell. In this role, the expression of c-Fos is transient and is a response to external stimuli.^11, 12^ c-Fos is required for normal development^11, 13^ and is involved in programmed cell death, though this role appears to vary by cell type.^14, 15^ All of these known roles of c-Fos indicate that it could have an important role in the renewal process of the peripheral taste cells. Using a conditional c-Fos knockout mouse, we found that selectively knocking out c-Fos expression in taste buds caused a degeneration of their structure due to a reduction in both cell proliferation and an increase in apoptosis. Our data identify a new role for c-Fos as a critical regulator of cell maintenance which is unique from its previously identified roles in other cell types.

## Results

### Global mRNA expression changes between 2 and 6-month-old mouse taste cells

Our current understanding of the processes that regulate taste cell renewal is limited by the lack of a comprehensive analysis of the genome-wide transcriptional changes that occur in the taste cells during the mouse lifespan. To identify the crucial factors involved in regulating taste cell renewal, we sought to use an unbiased approach of sequencing mRNA isolated from taste receptor cells of the CV/Fol taste papillae. As the taste cell renewal process declines with age,^16, 17^ we chose to identify the differences in gene expression levels between the taste cells of 2 and 6-month-old mice. By focusing on these age groups, we could identify factors that are affected in the taste cell renewal process before the consequences of aging are profound.^17, 18, 19, 20, 21^

**Figure 1:**
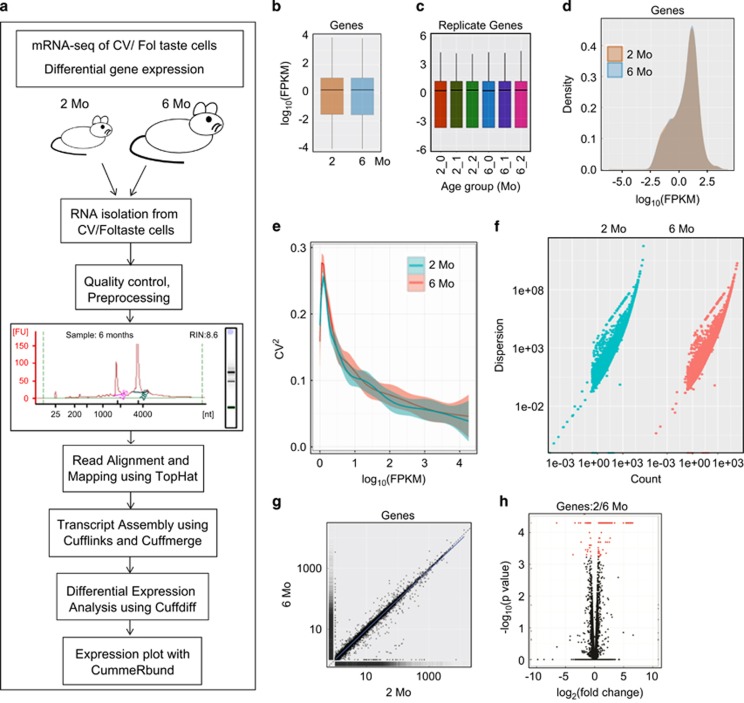
RNA-sequencing analysis of mouse CV/Fol taste cells from 2 and 6-month-old mice. (**a**) Schematic of RNA-sequencing workflow. Bioanalyzer profile of one of the RNA samples isolated from 6 month mice CV/Fol taste receptor cells. An RNA Integrity Number (RIN)⩾8 is recommended for RNA-sequencing library preparation. (**b**) Box plots of the expression RNA-seq read counts (FPKM) at the gene level and (**c**) the replicate genes level in CV/Fol taste cell RNA between 2 and 6-month-old mice taste cells. (**d**) Density plot shows the distribution of the FPKM values at the gene level in 2 and 6 month mice taste samples. (**e**) Variance of genes in 2 and 6-month-old mice taste samples. (**f**) Dispersion plots of 2 and 6-month-old mice taste samples. (**g**) Scatter plot shows differences in gene expression between 2 and 6-month-old mice taste cells. (**h**) Volcano plot identifying differentially expressed genes between 2 and 6-month-old mice taste samples (red is significant). Plots were generated using CummeRbund package v. 2.0

We performed a genome-wide transcriptome analysis through RNA-sequencing of CV/Fol taste cells isolated from C57Bl/6 mice belonging to different age groups (2 and 6 months). Isolated taste cells were collected from multiple mice and pooled together. Each replicate had taste cells isolated from 3–4 mice. Three such pooled experimental replicates were used for the RNA analysis. This provided sufficient RNA that no amplification of the mRNA was needed which reduces any potential bias that occurs due to an RNA amplification step. Figure 1a depicts the workflow of RNA-sequencing analysis performed to study the differential gene expression pattern of 2 and 6 month CV/Fol taste cells. Before sequencing, the quality of RNA was evaluated using a Bioanalyzer profile to ensure degradation had not occurred. The raw RNA-sequencing data were analyzed using the Tuxedo protocol (TopHat, Cufflinks and Cuffdiff) followed by visualization using CummeRbund. The RNA-seq read counts (FPKM) at the gene and the replicate genes level between 2 and 6-month-old mice CV/Fol taste cells were plotted as box-plots and show the normalized expression values (Figures 1b and c). These data demonstrates that the samples were comparable in their overall variability which increases our confidence that the differences detected were not due to skewed data. A density plot evaluates the quality of normalized distributions of FPKM scores across the analyzed samples which also show no difference in the distributions of the data sets (Figure 1d). The quality of RNA-sequencing data was further evaluated to assess the cross-replicate variability by measuring the variance of genes in 2 and 6-month-old mice taste samples (Figure 1e) and to rule out any bias due to over-dispersion for the two data sets (Figure 1f). As the data sets are comparable, we know that the differences that have been detected are not due to inherent biases in the data set but are due to changes in the expression patterns between the two samples. The scatter plot (Figure 1g) and the volcano plot (Figure 1h) identifies the global changes and trends in gene expression between 2 and 6-month-old mice taste samples. The data points displayed in red are significantly differentially expressed genes.

**Figure 2:**
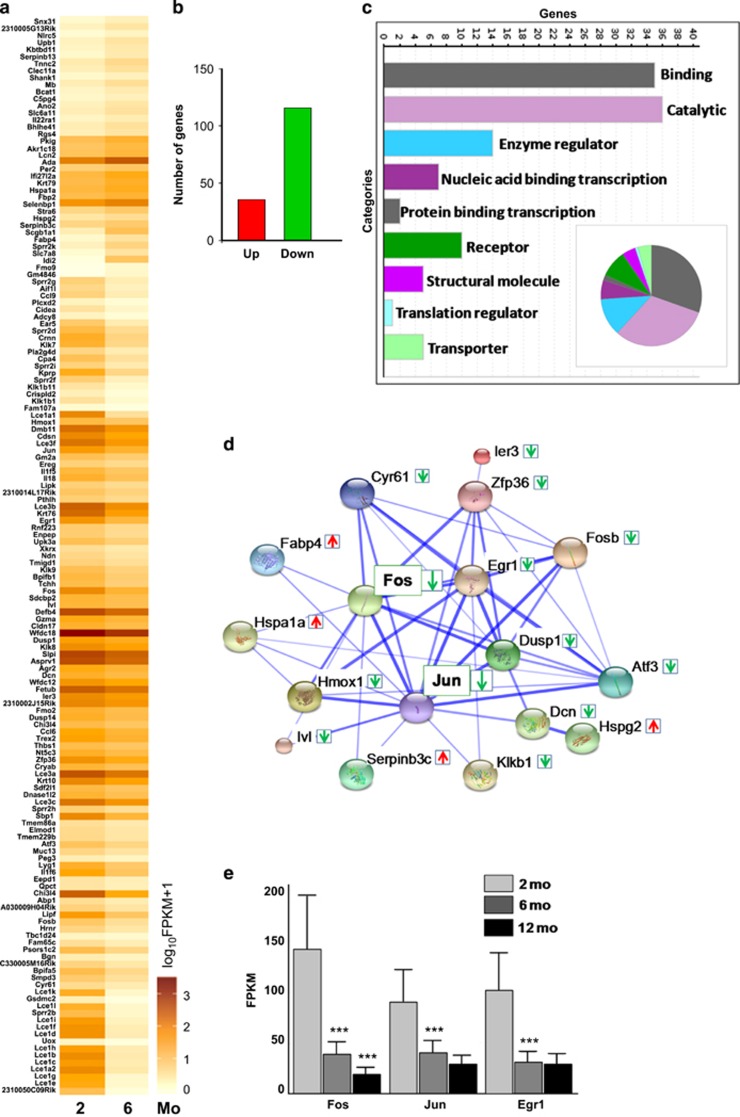
Multiple genes are differentially expressed in taste cells between 2 and 6 month age. (**a**) Heat map of differentially expressed genes in 2 and 6 month (Mo) mice taste cells. Higher expression is shown with darker color. (**b**) Plot shows the number of up- and downregulated genes in 6-month-old mouse taste cells. (**c**) Gene Ontology based classification of differentially expressed genes between 2 and 6 month mice. This analysis was performed using the Panther gene list analysis, molecular function. Bar graph depicts the number of genes associated with each functional category. The gene categories are also shown as pie chart (Inset). (**d**) String network analysis shows strong connections of AP1 transcription factors and potential target genes. The differentially expressed genes (see green/red arrows) in 6 month CV/Fol taste cells are shown in the ‘confidence view' of the string network analysis. Stronger associations are represented by thicker blue lines. (**e**) Relative FPKM values for c-Fos, c-Jun and Egr1 gene expression in 2, 6 and 12-month-old mice taste samples. Error bars denote s.d. of three independent experiments. c-Fos, 2–6 months (*P*<5.00E-05), 6–12 months (<5.00E-05), 2–12 months (<5.00E-05), c-Jun, 2–6 months (<5.00E-05), 6–12 months (NSD), 2–12 months (<5.00E-05), Egr1, 2–6 months (<5.00E-05), 6–12 months (NSD) 2–12 months (<5.00E-05). ***P<0.001

This approach identified 152 genes (of ~23 000 genes analyzed), with expression profiles significantly different between the 2 and 6 month samples. These differences are depicted in the heat map analysis (Figure 2a). Although the majority of the altered genes had reduced expression by 6 months, some genes were significantly upregulated (Figure 2b). Transcripts of all the known taste specific genes were detected while olfactory receptors were absent, confirming a taste specific transcription readout. Potential candidate genes were analyzed for the associated gene ontology which gave us insight on their roles in the regulation of adult taste function (Figure 2c). The gene ontology categorized the differentially expressed genes based on their known roles in other cell types. Most of the genes affected are involved in routine cell functions such as binding to calcium, RNA or protein, as well multiple enzymes and their regulators. Although these genes are needed for taste cell function, it is unlikely that they are the regulatory factors that control the processes involved in taste cell maintenance.

Quite unexpectedly, the AP1 transcription factors c-Fos, c-Jun and Fosb were significantly downregulated in the 6 month sample. String network analysis of the RNA-seq data identified several differentially regulated genes that are associated with c-Fos and c-Jun and suggests a potential relationship in taste cells (Figure 2d). Analysis of c-Fos expression in taste cells from 12-month-old mice found a further reduction in the mRNA expression levels with age (Figure 2e) while the levels of another candidate gene Egr1 (ref. 22) and c-Jun did not show a further change between the 6 and 12 month samples, though there is a significant reduction between 2 and 12 month samples for all three genes. Thus c-Fos is consistently and selectively downregulated in the taste buds of older mice. Taken together, these data suggest a potential role for the AP1 transcription factors in the maintenance of the peripheral taste system.

### AP1 transcription factors are downregulated in CV/Fol taste cells from 6-month-old mice

**Figure 3:**
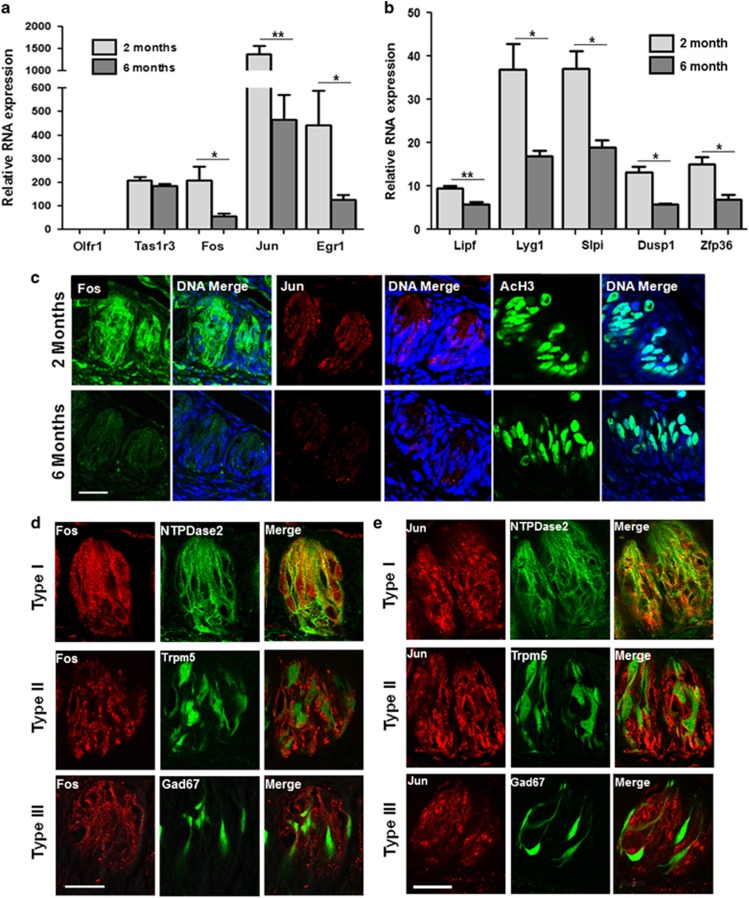
AP1 transcription factors downregulate in 6-month-old CV/Fol taste cells. (**a**) qRT-PCR analysis of the relative expression for different genes in CV/Fol taste cells from 2 and 6 month mice: c-Fos (*P*=0.033), c-Jun (*P*=0.0067), Egr1 (*P*=0.045). Taste cell receptor gene (Tas1r3, *P*>0.05), and olfactory gene (Olfr1, *P*>0.05). The olfactory-specific gene (Olfr1) is not expressed in taste cells and was used as a negative control. (**b**) qRT-PCR validation of selected genes: Lipf (*P*=0.0091), Lyg1 (*P*=0.0293), Slpi (*P*=0.0144), Dusp1 (*P*=0.011) and Zfp36 (*P*=0.0133) in 2 and 6 month mice. Statistical significance was determined using Student's *t* test. Error bars denote S.D. of three independent experiments. (**c**) Immunohistochemical analysis shows reduced labeling for c-Fos, c-Jun and acetylated histone H3 (AcH3) in CV taste bud in 6-month-old mouse compared with 2-month-old mice. Bar=20 *μ*M. (**d**) Localization of c-Fos (red) and type I (NTPdase2, green), type II (Trpm5-GFP) and type III (GAD67-GFP) markers in CV taste papillae from 2-month-old mice. Nuclei were labeled with DAPI (blue). (**e**) Similarly, the localization pattern of c-Jun and different taste cell type markers. Bar=20 *μ*M. *P<0.05; **P<0.01

We validated the differences in the expression of c-Fos, c-Jun and other selected candidate genes from the RNA-sequencing data set using qRT-PCR and found a strong correlation with the RNA-sequencing results. We evaluated the taste receptor gene (Tas1r3) expression as a taste specific control and found it was unchanged. As a negative control, we confirmed that olfactory-specific gene (Olfr1) is not expressed in taste cells (Figure 3a). We also selected several genes to further validate their RNA-seq differential expression pattern. Genes having enzymatic activity such as Lipf (lipase F), Lyg1 (Lysozyme G-Like 1), Slpi (secretory leukocyte protease inhibitor) and Dusp1 (Dual specificity protein phosphatase 1) were tested along with Zfp36 (zinc finger protein 36 homolog) which has a role in inflammatory responses. Out of these genes, Dusp1 is an immediate early gene like the AP1 factors c-Fos and c-Jun.^23^ The RNA-sequencing and subsequent qRT-PCR analyses found these genes to be significantly downregulated in 6-month-old taste cells (Figure 3b).

Immunohistochemical analysis of the CV papillae found that the protein expression of both c-Fos and c-Jun was reduced in the 6-month-old mice compared with the 2-month-old mice. However, the level of acetylated histone H3 (a marker of transcriptionally active chromatin) was similar in both the age groups suggesting that transcriptional activity is not different between 2 and 6-month-old taste cells (Figure 3c). Taste buds contain three major types of taste cells that can be differentiated based on the expression of specific markers.^24, 25, 26^ Immunohistochemical analysis of the CV papillae revealed that c-Fos and c-Jun are widely expressed and have overlapping expression with the type I marker, NTPDase (Figures 3d and e). Very little overlap with Type II cells (assessed using TRPM5-GFP expression, c-Fos: 10% overlap, c-Jun:16%) and almost no overlap with the type III marker (assessed using GAD-GFP expression, c-Fos: 5% overlap, c-Jun: 5% overlap) was found. These data indicate that the AP1 transcription factors are mostly expressed in type I cells. Due to the anatomy of some type I cells and their tendency to wrap around other cell types, the small overlap we measured for c-Fos and c-Jun could be attributed to the type I cells orientation with the type II and type III cells. We cannot rule out that a small percentage of types II and III cells do express c-Fos or c-Jun but it does not appear to be a significant portion of these taste cells. Interestingly, we observed that c-Fos and c-Jun have a diffused localization in the taste bud cells (both nuclear and cytoplasmic). It is possible that these AP1 factors have some distinct non-transcriptional roles in mature taste cells as has been shown in other tissues.^27, 28^

### c-Fos knockout alters taste bud structure

**Figure 4:**
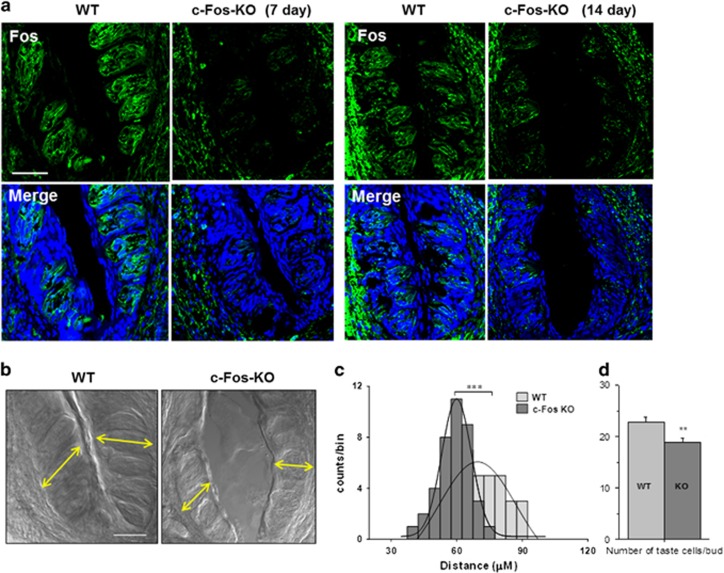
Loss of c-Fos alters of taste bud structure. (**a**) c-Fos expression is reduced by 7 and 14 days post-tamoxifen treatment in c-Fos-KO taste cells. Immunohistochemical analysis shows reduced labeling for c-Fos (green) in CV taste papillae from a tamoxifen-treated c-Fos-KO mice compared with control mice. Bar=50 *μ*M. (**b**) DIC image shows changes in the structural integrity of CV taste papillae in tamoxifen-treated c-Fos-KO mice after 14 days (right panel) compared with control mice (left panel). Bar=50 *μ*M. (**c**) A distribution histogram of distances between the base and the tip of individual CV taste buds (see yellow arrows in **b**). WT and c-Fos-KO distance distributions were significantly different at 95% confidence level (non parametric Mann–Whitney test; ***P=0.0001). (**d**) Analysis of the number of taste receptor cells/bud in c-Fos-KO mice found a significant reduction in the number of taste cells/bud compared with controls (**P<0.01, WT=14 buds; KO=15 buds)

Based on the known functions of AP1 transcription factors in cell growth,^8^ we predicted that the reduced protein expression of c-Fos and c-Jun affects the taste cell renewal process. As c-Fos and c-Jun work together, we generated a conditional knockout mouse of c-Fos gene using an inducible Cre-lox system. Tamoxifen-inducible Cre-Keratin 14 (Cre-K14) expresses predominantly in lingual epithelium and regenerating taste cells and has been successfully used to knockout genes of interest in taste buds.^29, 30^ The mice were analyzed for c-Fos expression in both wild type (WT) and c-Fos-KO mice 7 and 14 days after the final tamoxifen injection. Immunofluorescence analysis of c-Fos-KO mice showed drastically reduced c-Fos expression compared with WT mice after 7 and 14 days (Figure 4a). c-Fos was lower but not absent in the c-Fos KO tongues, which is consistent with the findings of Okubo *et al.*^29^ that the K14CreER lineage tagging results in mosaic labeling, and thus with incomplete deletion of c-Fos.

Even with some residual c-Fos expression in the CV papillae, the loss of c-Fos had a substantial effect on the structure of the CV papillae. Compared with WT mice, the size of the individual CV taste buds were significantly reduced in the c-Fos-KO mice (Figure 4b, compare the length of yellow arrows) and the structure of the CV papillae began to break down. The differences in the taste bud size between WT and c-Fos-KO mice were quantitated (*n*=5 mice each) using all the taste buds from a single optical section located in the middle of the CV papillae. These data are plotted as a distribution histogram and reveal a significant reduction in the size of the taste buds from c-Fos-KO mice (Figure 4c). Cell counting of the number of taste receptor cells/bud in the c-Fos-KO mice compared with the WT revealed a small but significant decrease in the number of taste cells/bud in the c-Fos-KO mice, 14 days after treatment (*n*=14 buds for WT and 15 buds for KO, *P*<0.01, Figure 4d). Cell counting of taste receptor cells/bud 7 days after treatment revealed no significant difference in the number of taste cells/bud (*n*=11 for WT and 11 for KO, *P*=0.447). Thus, AP1 transcription factors are required to maintain normal taste bud structure and when c-Fos expression is lost, the peripheral taste structures begin to break down.

### c-Fos regulates taste cell turnover

**Figure 5:**
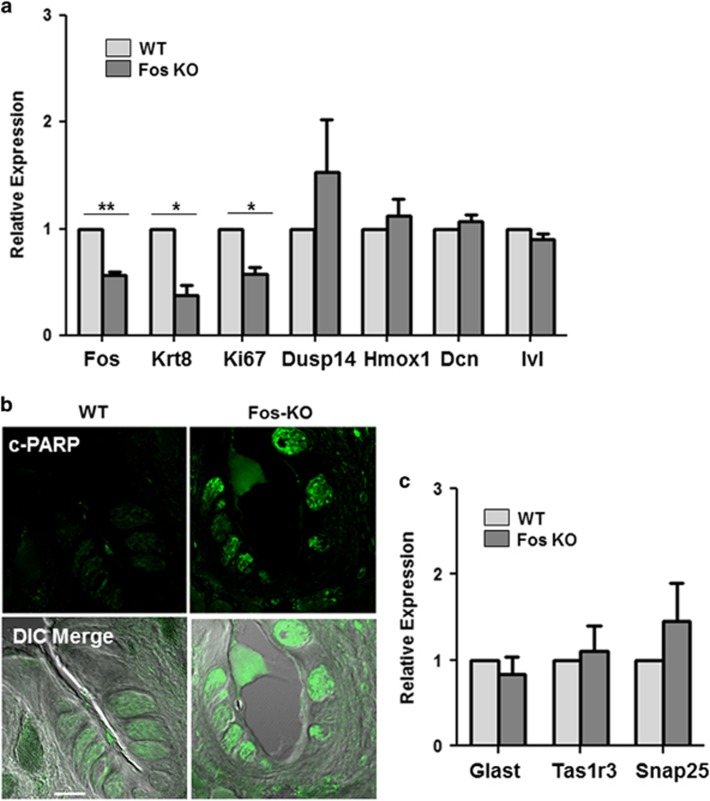
Loss of c-Fos results in reduced taste cell differentiation and increased apoptosis. (**a**) Relative expression of indicated genes: c-Fos (*P*=0.0056), Krt8 (*P*=0.0252), Ki67 (*P*=0.0202), Dusp14, Hmox1, Dcn and Ivl in CV/Fol taste cells from WT and c-Fos-KO mice. Statistical significance was determined using Student's *t* test. Error bars denote S.D. of three independent experiments. (**b**) Immunohistochemical analysis shows the expression level of cleaved-PARP in the CV taste cells of WT and c-Fos-KO mice, 14 days after tamoxifen treatment. (**c**) qRT-PCR analysis of taste cell type markers Glast (type I), Tas1R3 (type II) and Snap25 (type III). Error bars denote S.D. of three independent experiments. *P<0.05; **P<0.01

Using qPCR, we evaluated the mRNA levels of c-Fos after the mice were treated with tamoxifen. We found significantly reduced but not abolished c-Fos mRNA in taste cells after the tamoxifen treatment (Figure 5a). Based on our immunohistochemical analysis (Figure 4a), most of the c-Fos protein expression is gone, but some residual protein may remain. However, this low level of c-Fos was not sufficient to maintain the taste cells in their normal configuration. We measured the mRNA levels of several genes found in the string network with c-Fos in the c-Fos-KO mouse. These include Hmox1 (heme oxygenase 1), Ivl (Involucrin), Dusp14 (Dual Specificity Phosphatase 14) and Dcn (Decorin) which are known to be involved in a diverse array of cellular processes and reported to be modulated by c-Fos.^31, 32, 33, 34^ Analysis of the mRNA levels for these target genes found no reduction in their expression after loss of c-Fos even though their levels were reduced in the RNA-sequencing analysis. These data suggest that the low levels of c-Fos that remain after knock down are sufficient to maintain the normal expression levels of these genes or their expression levels are not directly dependent on c-Fos activity. It is also possible that the cells have compensatory mechanisms to maintain their expression when c-Fos is lost. Further experiments will be needed to determine which of these hypotheses are correct.

The altered morphology of the taste buds suggests that c-Fos plays a critical role in taste cell renewal, taste cell death or both processes. To explore the effect of c-Fos deletion on taste bud differentiation, we compared the expression levels of Keratin 8 (Krt8) in WT and c-Fos-KO mice taste cells. Krt8 is a differentiation marker expressed in taste cells that have been terminally differentiated.^35^ The level of Krt8 was significantly reduced in the c-Fos-KO taste cells suggesting that cell differentiation is impaired when c-Fos expression is reduced (Figure 5a). We also analyzed cell proliferation by measuring the expression levels of the proliferation marker Ki67 (ref. 36) in WT and c-Fos-KO taste cells (Figure 5a). Loss of c-Fos resulted in a significant reduction of Ki67 expression.

Studies have shown that c-Fos is also an important regulator of cell survival.^37, 38^ To determine if taste cell survival is affected by the loss of c-Fos, we analyzed the apoptosis levels in taste cells. Poly (ADP-ribose) polymerase-1 (PARP-1) is one of the cellular substrates of caspases and the cleavage of PARP-1 is considered an important early marker of apoptosis.^39, 40^ Using an Anti-PARP p85 Fragment antibody that has been shown to be a reliable immunohistochemical marker for the specific detection of apoptotic cells,^41, 42^ we found that the level of apoptosis significantly increased in the c-Fos-KO taste cells compared with WT (as indicated by higher cleaved-PARP intensity in Figure 5b). Thus c-Fos acts to prevent taste cell death as well as to promote taste cell renewal.

Interestingly, the mRNA expression of different taste cell type markers did not change after c-Fos was removed within the time points tested (Figure 5c). The expression levels of the taste cell markers were also unchanged in our RNA-sequencing data set. Protein expression levels of another type II taste cell marker, PLCβ2, also did not change in the c-Fos-KO mice compared with controls (37% expression in WT, 39% expression in KO, *P*>0.05). Thus, while the terminal differentiation of taste cells may be affected in c-Fos KO mice (as seen by the reduction in Krt8 expression), the cell type markers that we tested were not reduced within the timeframe of these experiments. While surprising, these data do agree with the findings of Gaillard and Barlow^6^ that showed comparable levels of cell type markers in young (2 mo) and older (6 mo) old mice, even while Wnt signaling was significantly reduced. Clearly, the cellular processes controlling taste cell function are complex and further studies will be needed to connect the signaling pathways that control taste cell maintenance with the expression of taste-specific genes.

## Discussion

Although it is clear that taste cell renewal has significant effects on taste function, the precise molecular pathways and factors that regulate this crucial taste cell property are poorly understood. An earlier study found that taste bud size and taste cell density decreases in humans with age but the molecular mechanisms involved were not identified.^43^ Our RNA-sequencing analysis has provided a global picture of taste-specific gene expression changes that occur within a span of a few months in adult mice. We found significant differences in the expression of multiple genes (~152) in the peripheral taste receptor cells of 6-month-old mice compared with young adult (2 month old) mice. Most of these genes have roles in the normal function of taste cells and are unlikely to be involved in the processes that control taste cell turnover and renewal. However, our bioinformatics analyses identified several candidate factors that have the potential to affect taste cell renewal. The most striking of these candidates were the AP1 transcription factors, c-Fos and c-Jun whose expression consistently and progressively decreased from 2 to 12-month-old mice taste buds. Indeed, c-Fos and c-Jun are central to a network of genes that are differentially expressed from 2 to 6 month mice taste cells (Figure 2) through their function as transcriptional regulators. Taken together, these data support the idea that the AP1 transcription factors and their associated genes are important for the maintenance of the peripheral taste system.

The Wnt/β-catenin signaling pathway has been shown to regulate adult taste cell renewal^6^ and β-catenin activity is significantly reduced in the taste buds by 6 months. Several studies have shown an association between AP1 and β-catenin in the regulation of diverse gene targets in other tissues. In these instances, AP1 factors are downstream targets of the Wnt/β-catenin pathway.^44, 45^ It is possible that AP1 factors co-operate with the Wnt/β-catenin signaling pathway in co-regulating the taste cell renewal process or are downstream targets for this pathway.

c-Fos has been used as an activity indicator in taste cells in the context of inflammation signaling^46, 47^ and it is commonly used as an activity indicator in neurons.^22^ Indeed, c-Fos is widely used as an activity indicator in many studies measuring taste induced activity in the central taste system.^48, 49, 50^ A spike in c-Fos immunoreactivity in the central amygdala and insular cortex regions of brain was observed during taste aversion conditioning and on introduction of novel tastants.^51^ Other work reported that c-Fos KO animals have deficits in the normal long-term learning and memory that is normally associated with aversive taste learning.^52^ To our knowledge, however, the role of c-Fos in taste receptor cells has not previously been evaluated. Our data suggest that c-Fos is much more than an activity indicator in peripheral taste cells.

Our data reveal that a significant reduction in c-Fos causes a loss in the structural integrity of taste buds and accelerates the deterioration of taste papillae structure. Further analysis of our RNA-sequence data set found that the pathways required for general epidermal differentiation and cornification are also downregulated in the 6 month samples. These genes are responsible for maintaining the normal epidermal surface which includes taste buds. Break down in the epidermal surface causes an increase susceptibility to infections and an upregulation in inflammatory cytokines.^53^ Analyses of the co-expression of c-Fos and c-Jun with taste cell markers reveal that these two proteins are primarily, even perhaps exclusively, expressed in type I cells. Thus the loss of taste cells that occurs in the c-Fos KO mice 14 days after tamoxifen activation are likely to be primarily type I cells. Indeed, we found no loss of type II cells in the c-Fos KO mice compared with controls.

Type I taste cells comprise ~50% of a taste bud and have properties similar to glial cells in the nervous system. These taste cells can wrap around other taste cell types and express proteins involved in the deactivation and reuptake of neurotransmitters from the surrounding milieu.^54, 55, 56^ These characteristics have supported the hypothesis that type I cells function primarily to support the other transducing taste cells. However, another study determined that type I cells express amiloride sensitive sodium channels which are involved in salt transduction^57^ suggesting that the type I taste cells likely also transduce salt stimuli. Our data reveal that the AP1 factors are expressed primarily in the type I cells and appear to be important for the functional integrity of the taste cells and its surrounding epithelium. These findings agree with the idea that type I cells primarily function to maintain the functional integrity of the taste bud.

Our data has identified a critical role for c-Fos in the routine maintenance of taste cells, and this role may be common to multiple systems. The results reveal that c-Fos is required to maintain the structural integrity of taste buds by affecting both taste cell renewal and apoptosis. In conclusion, our data have identified a new role for AP1 transcription factors in taste cell maintenance and provides new insights into the current understanding of the molecular basis of the taste cell renewal.

## Materials and Methods

### Taste cell isolation

Animals were cared for in compliance with the University at Buffalo Animal Care and Use Committee (IACUC). For generating knockouts, we used the following mouse lines: Tg(KRT14-cre/ERT)20Efu/J, stock no: 005107 and B6;129-Fos^tm1Mxu^/Mmjax, stock no: 024767, The Jackson Laboratory. Isolated taste receptor cells were collected from adult mice (at ages 2, 6 or 12 months) following previously described procedures.^58, 59, 60, 61, 62, 63, 64, 65^ Taste buds were harvested from CV and Fol papillae of C57BL/6 mice (*n*=3 mice per sample for older mice, 4 mice per sample for 2 mo mice). Mice were killed with CO_2_ and cervical dislocation. Tongues were removed and then injected under the lingual epithelium with 100 *μ*l of an enzymatic solution containing 0.7 mg of collagenase B (Roche, Indianapolis, IN, USA), 3 mg of dispase II (Roche), and 1 mg of trypsin inhibitor (Sigma, St. Louis, MO, USA) per milliliter of Tyrode's solution (140 mM NaCl, 5 mM KCl, 1 mM MgCl_2_, 3 mM CaCl_2_, 10 mM Hepes, 10 mM glucose, and 1 mM pyruvic acid, pH 7.4). Tongues were incubated with oxygenated Tyrode's solution for 20 min before the epithelial layer was peeled off and incubated in Ca^2+^ free Tyrode's solution (140 mM NaCl, 5 mM KCl, 10 mM Hepes, 2 mM BAPTA, 10 mM glucose and 1 mM pyruvic acid, adjusted to pH 7.4) for 30 min. The tissue was put back into Tyrode's and Fol/CV taste cells were harvested with a capillary pipette using gentle suction. All the taste cells from each Fol and CV papillae were collected into Tyrode's on ice. For each sample, cells from multiple mice were pooled together. It took ~10 min to collect all the cells from three mice for one pooled sample. The cells were centrifuged and the Tyrode's solution was completely removed. The pelleted cells were stored at −80 ^o^C until the RNA was isolated.

### Library construction and sequencing for RNA-Seq

Total RNA was isolated using the Nucleospin RNA XS kit (Clontech, Mountain View, CA, USA) from the pooled samples of taste cells of 2 mo and 6-month-old mice at the same time. Triplicate samples of the isolated taste cells from 12-month-old mice (*n*=3 mice per replicate) were isolated and analyzed at a later date. One of the replicates of the 12 month samples was of lower quality so we did not do a full analysis of the 12 month samples. RNA samples for three replicate experiments (pooled taste cells isolated from 3 to 4 mice for each replicate experiment) per age group were quantified using Ribogreen Assay (Invitrogen, Carlsbad, CA, USA). The quality of samples was checked using Bioanalyzer 2100 RNA nano 6000 chip (Agilent, Santa Clara, CA, USA). The TruSeq RNA sample preparation kit (Illumina, San Diego, CA, USA) was used to prepare cDNA libraries from RNA samples. Samples were poly A selected to isolate mRNA, the mRNA was cleaved into fragments, the first strand reverse transcribed to cDNA using SuperScript II Reverse Transcriptase (Invitrogen) and random primers, followed by second strand cDNA synthesis using Second Strand Master Mix supplied with the kit. After end repair, the addition of a single ‘A' base, and ligation with adapters, the products were enriched and purified with PCR to create the final cDNA library as per manufacturer's protocol. cDNA libraries were quantified using Picogreen Assay (Invitrogen) and Library Quantification kit (Kapa Biosystems, Wilmington, MA). Agilent Bioanalyzer 2100 DNA 7500 chip is used to confirm the quality and size of the cDNA libraries. The cDNA libraries were then normalized, pooled and sequenced using the Illumina HiSeq2500 following the manufacturer's instructions at the UB Genomics and Bioinformatics Core Facility, Buffalo, NY, USA.^66, 67^

Raw sequencing reads (obtained from the six sets of mice taste cell RNA-Seq experiments (three for taste cells from 2-month-old mice and 3 for taste cells from 6 month old mice) were mapped to the *Mus musculus* genome (GRCm38/mm10 build) using TopHat (v2.0.7), with default parameters and Illumina's iGenomes transcript annotation file ‘genes.gtf' (from RefSeq; mm10) available at http://support.illumina.com/sequencing/sequencing_software/igenome.ilmn. This data set is available in the NCBI GEO database, accession number GSE85308. Gene isoform level transcript abundances were quantified as Fragments Per Kilobase of transcript per Million mapped reads (FPKM) using Cufflinks (v2.1.1). Differentially expressed genes information was calculated using Cuffdiff and the plots were visualized using CummeRbund package v. 2.0 (Boston, MA, USA).

### Tamoxifen treatment

The mutant (c-Fos-KO) and WT mice had similar growth and breeding behavior in the absence of any tamoxifen treatment. At the age of 6–8 weeks, c-Fos-KO and WT mice were administered tamoxifen (75 mg/Kg body weight) via intraperitoneal injection (using an IACUC approved procedure) once every 24 h for 6 consecutive days. Both the KO and the WT mice were monitored for any adverse effects. Mice were euthanized and tongues were collected either 7 or 14 days post injection.

### Immunohistochemistry

Immunohistochemical analysis was performed as previously described.^58, 68^ Antibodies were: anti-cleaved PARP (ab4830; 1:50), anti-c-Fos (ab7963; 1:50), anti-c-Jun (ab31419; 1:50), anti-AcH3 (ab1791; 1:200) from Abcam and anti-NTPdase2 (1:100).^69^ Secondary antibodies were purchased from Jackson ImmunoResearch (West Grove, PA, USA). Some experiments were performed with anti-c-Fos that had a fluorophore directly attached using the Mix-n-Stain CF Dye antibody labeling kit (Biotium, Hayward, CA, USA). The images were taken via LSCM and we have used single optical section to demonstrate double labeling as well as for cell counting analyses. Identical settings for laser intensity and brightness/contrast were used for comparative analysis between sections.

### RNA analysis

Taste buds/cells were isolated from individual mice. Each replicate consisted of isolated taste cells pooled from 2 to 3 mice. Three such pooled experimental replicates were used for the qRT-PCR analysis. Total RNA was prepared using the Nucleospin RNA XS kit (Clontech) and cDNA was prepared using the Bio-Rad cDNA synthesis kit (Hercules, CA, USA). Primers for RNA analysis are listed in Table 1. All experiments consisted of at least three biological repeats. Data for the qPCR was normalized to GAPDH and were plotted as average values with S.D.

## Figures and Tables

**Table 1:** Primers used for qRT-PCR analyses

*Gene name*	*Primer sequence 5′–3′*
mFos FP	GGTTTCAACGCCGACTACGA
mFos RP	GCGCAAAAGTCCTGTGTGTT
mJun FP	CGACCTTCTACGACGATGCC
mJun RP	AGAAGGTCCGAGTTCTTGGC
mEGR1FP	TGAGCATGACCAATCCTCCG
mEGR1RP	CAGGGATCATGGGAACCTGG
mTas1R3 FP	AGGCCACTCTCAACCAGAGA
mTas1R3 RP	GGGAGCAAGGCAGATCCATT
mOlfr1 FP	TGGCCAGCATCTTTCTTGTCC
mOlfr1 RP	CCAGAGCCCCCTTTATGTCTC
mLipf FP	CGAGGGAATACATGGTCCCG
mLipf RP	AAACCGATAGTGGTGCCCTG
mLyg1 FP	GCAGTTGGGGATGCTATGGA
mLyg1 RP	GGACTCCGCAATAGGTCAGG
mSlpi FP	AAGCCACAATGCCGTACTGA
mSlpi RP	CACACTGGTTTGCGAATGGG
mDusp1 FP	AGTGCCTATCACGCTTCTCG
mDusp1 RP	CCTCCACAGGGATGCTCTTG
mZfp36 FP	GGACCTACTCAGAAAGCGGG
mZfp36 RP	ACTTGTGGCAGAGTTCCGTT
mki67 FP	ATCCAGCTGCCTGTAGTGTC
mki67 RP	AGGCCCTTGGCATACACAAA
mKrt8 FP	CGGGGGATCCAACACTTTCA
mKrt8 RP	GCTTCCCATCTCGGGTTTCA
mSnap25 FP	TCGATCGTGTCGAAGAAGGC
mSnap25 RP	AGGCCACAGCATTTGCCTAA
mGlast FP	CTGGTAACCCGGAAGAACCC
mGlast RP	GGGGAGCACAAATCTGGTGA

## Abbreviations

AP1: Activator protein-1
CV: circumvallate
Dcn: Decorin
Dusp1: Dual specificity protein phosphatase 1
Dusp14: Dual specificity phosphatase 14
EGR1: Early growth response 1
Fol: Foliate
FPKM: Fragments per kilobase of transcript per million mapped reads
GAPDH: Glyceraldehyde phosphate dehydrogenase
Hmox1: Heme oxygenase 1
K14: Keratin 14
KO: Knockout
Lipf: Lipase F
Ivl: Involucrin
Lyg1: Lysozyme G-like 1
PARP-1: Poly (ADP-ribose) polymerase-1
qPCR: Quantitative polymerase chain reaction
RT-PCR: Reverse transcription polymerase chain reaction
Shh: Sonic hedgehog
Slpi: secretory leukocyte protease inhibitor
Wnt: Wingless-related integration site
WT: Wild type
Zfp36: Zinc-finger protein 36 homolog

## References

[bib1] Grando LJ, Mello AL, Salvato L, Brancher AP, Del Moral JA, Steffenello-Durigon G. Impact of leukemia and lymphoma chemotherapy on oral cavity and quality of life. Spec Care Dentist 2015 (doi:10.1111/scd.12113). 10.1111/scd.12113 25963973

[bib2] Roura E, Foster S, Winklebach A, Navarro M, Thomas W, Campbell K et al. Taste and hypertension in humans: targeting cardiovascular disease. Curr Pharm Des 2016; 22: 2290–2305. 26881437 10.2174/1381612822666160216151545

[bib3] Mukherjee N, Delay ER. Cyclophosphamide-induced disruption of umami taste functions and taste epithelium. Neuroscience 2011; 192: 732–745. 21782899 10.1016/j.neuroscience.2011.07.006

[bib4] Barlow LA, Klein OD. Developing and regenerating a sense of taste. Curr Top Dev Biol 2015; 111: 401–419. 25662267 10.1016/bs.ctdb.2014.11.012 PMC4435577

[bib5] Liu HX, Ermilov A, Grachtchouk M, Li L, Gumucio DL, Dlugosz AA et al. Multiple Shh signaling centers participate in fungiform papilla and taste bud formation and maintenance. Dev Biol 2013; 382: 82–97. 23916850 10.1016/j.ydbio.2013.07.022 PMC3968530

[bib6] Gaillard D, Barlow LA. Taste bud cells of adult mice are responsive to Wnt/beta-catenin signaling: implications for the renewal of mature taste cells. Genesis 2011; 49: 295–306. 21328519 10.1002/dvg.20731 PMC3536498

[bib7] Iwatsuki K, Liu HX, Gronder A, Singer MA, Lane TF, Grosschedl R et al. Wnt signaling interacts with Shh to regulate taste papilla development. Proc Natl Acad Sci USA 2007; 104: 2253–2258. 17284610 10.1073/pnas.0607399104 PMC1794217

[bib8] Hess J, Angel P, Schorpp-Kistner M. AP-1 subunits: quarrel and harmony among siblings. J Cell Sci 2004; 117: 5965–5973. 15564374 10.1242/jcs.01589

[bib9] Eckert RL, Adhikary G, Young CA, Jans R, Crish JF, Xu W et al. AP1 transcription factors in epidermal differentiation and skin cancer. J Skin Cancer 2013; 2013: 537028. 23762562 10.1155/2013/537028 PMC3676924

[bib10] Mehic D, Bakiri L, Ghannadan M, Wagner EF, Tschachler E. Fos and jun proteins are specifically expressed during differentiation of human keratinocytes. J Invest Dermatol 2005; 124: 212–220. 15654976 10.1111/j.0022-202X.2004.23558.x

[bib11] Velazquez FN, Caputto BL, Boussin FD. c-Fos importance for brain development. Aging (Albany NY) 2015; 7: 1028–1029. 26684501 10.18632/aging.100862 PMC4712328

[bib12] Lamprecht R, Dudai Y. Transient expression of c-Fos in rat amygdala during training is required for encoding conditioned taste aversion memory. Learn Mem 1996; 3: 31–41. 10456074 10.1101/lm.3.1.31

[bib13] Velazquez FN, Prucca CG, Etienne O, D'Astolfo DS, Silvestre DC, Boussin FD et al. Brain development is impaired in c-fos -/- mice. Oncotarget 2015; 6: 16883–16901. 26143639 10.18632/oncotarget.4527 PMC4621926

[bib14] Smeyne RJ, Vendrell M, Hayward M, Baker SJ, Miao GG, Schilling K et al. Continuous c-fos expression precedes programmed cell death *in vivo*. Nature 1993; 363: 166–169. 8483500 10.1038/363166a0

[bib15] Kalra N, Kumar V. c-Fos is a mediator of the c-myc-induced apoptotic signaling in serum-deprived hepatoma cells via the p38 mitogen-activated protein kinase pathway. J Biol Chem 2004; 279: 25313–25319. 15078869 10.1074/jbc.M400932200

[bib16] Feng P, Huang L, Wang H. Taste bud homeostasis in health, disease, and aging. Chem Senses 2014; 39: 3–16. 24287552 10.1093/chemse/bjt059 PMC3864165

[bib17] Shin YK, Cong WN, Cai H, Kim W, Maudsley S, Egan JM et al. Age-related changes in mouse taste bud morphology, hormone expression, and taste responsivity. J Gerontol A Biol Sci Med Sci 2012; 67: 336–344. 22056740 10.1093/gerona/glr192 PMC3410661

[bib18] Moore EM, RDt Forrest, Boehm SL 2nd. Genotype modulates age-related alterations in sensitivity to the aversive effects of ethanol: an eight inbred strain analysis of conditioned taste aversion. Genes Brain Behav 2013; 12: 70–77. 23171343 10.1111/gbb.12004 PMC3553292

[bib19] Fukunaga A. [Age-related changes in renewal of taste bud cells and expression of taste cell-specific proteins in mice]. Kokubyo Gakkai Zasshi 2005; 72: 84–89. 15856776 10.5357/koubyou.71and72.84

[bib20] Fukunaga A, Uematsu H, Sugimoto K. Influences of aging on taste perception and oral somatic sensation. J Gerontol A Biol Sci Med Sci 2005; 60: 109–113. 15741292 10.1093/gerona/60.1.109

[bib21] Zeng Q, Kwan A, Oakley B. Gustatory innervation and bax-dependent caspase-2: participants in the life and death pathways of mouse taste receptor cells. J Comp Neurol 2000; 424: 640–650. 10931486 10.1002/1096-9861(20000904)424:4<640::aid-cne6>3.0.co;2-n

[bib22] Minatohara K, Akiyoshi M, Okuno H. Role of immediate-early genes in synaptic plasticity and neuronal ensembles underlying the memory trace. Front Mol Neurosci 2015; 8: 78. 26778955 10.3389/fnmol.2015.00078 PMC4700275

[bib23] Sakai S, Ikematsu K, Matsuo A, Tsai CT, Nakasono I. Expression of C-fos, Fos-B, Fosl-1, Fosl-2, Dusp-1 and C-jun in the mouse heart after single and repeated chlorpromazine administrations. Leg Med 2010; 12: 284–288. 10.1016/j.legalmed.2010.07.005 20843724

[bib24] Barlow LA. Progress and renewal in gustation: new insights into taste bud development. Development 2015; 142: 3620–3629. 26534983 10.1242/dev.120394 PMC4647210

[bib25] Finger TE, Simon SA. Cell Biology of taste epitheliumFinger TE, Silver WL, Restrepo D. The Neurobiology of Taste and Smell. Wiley-Liss: New York, 2000: 287–314.

[bib26] Lindemann B. Receptors and transduction in taste. Nature 2001; 413: 219–225. 11557991 10.1038/35093032

[bib27] Caputto BL, Cardozo Gizzi AM, Gil GA. c-Fos: an AP-1 transcription factor with an additional cytoplasmic, non-genomic lipid synthesis activation capacity. Biochim Biophys Acta 2014; 1841: 1241–1246. 24886961 10.1016/j.bbalip.2014.05.007

[bib28] Malnou CE, Salem T, Brockly F, Wodrich H, Piechaczyk M, Jariel-Encontre I. Heterodimerization with Jun family members regulates c-Fos nucleocytoplasmic traffic. J Biol Chem 2007; 282: 31046–31059. 17681951 10.1074/jbc.M702833200

[bib29] Okubo T, Clark C, Hogan BL. Cell lineage mapping of taste bud cells and keratinocytes in the mouse tongue and soft palate. Stem Cells 2009; 27: 442–450. 19038788 10.1634/stemcells.2008-0611 PMC4337989

[bib30] Vasioukhin V, Degenstein L, Wise B, Fuchs E. The magical touch: genome targeting in epidermal stem cells induced by tamoxifen application to mouse skin. Proc Natl Acad Sci USA 1999; 96: 8551–8556. 10411913 10.1073/pnas.96.15.8551 PMC17554

[bib31] Florin L, Hummerich L, Dittrich BT, Kokocinski F, Wrobel G, Gack S et al. Identification of novel AP-1 target genes in fibroblasts regulated during cutaneous wound healing. Oncogene 2004; 23: 7005–7017. 15273721 10.1038/sj.onc.1207938

[bib32] Gong P, Stewart D, Hu B, Vinson C, Alam J. Multiple basic-leucine zipper proteins regulate induction of the mouse heme oxygenase-1 gene by arsenite. Arch biochem biophys 2002; 405: 265–274. 12220541 10.1016/s0003-9861(02)00404-6

[bib33] Efimova T, Eckert RL. Regulation of human involucrin promoter activity by novel protein kinase C isoforms. J Biol Chem 2000; 275: 1601–1607. 10636851 10.1074/jbc.275.3.1601

[bib34] Malik AN, Vierbuchen T, Hemberg M, Rubin AA, Ling E, Couch CH et al. Genome-wide identification and characterization of functional neuronal activity-dependent enhancers. Nat Neurosci 2014; 17: 1330–1339. 25195102 10.1038/nn.3808 PMC4297619

[bib35] Gaillard D, Xu M, Liu F, Millar SE, Barlow LA. β-Catenin signaling biases multipotent lingual epithelial progenitors to differentiate and acquire specific taste cell fates. PLoS Genet 2015; 11: e1005208. 26020789 10.1371/journal.pgen.1005208 PMC4447363

[bib36] Scholzen T, Gerdes J. The Ki-67 protein: from the known and the unknown. J Cell Physiol 2000; 182: 311–322. 10653597 10.1002/(SICI)1097-4652(200003)182:3<311::AID-JCP1>3.0.CO;2-9

[bib37] Shaulian E, Karin M. AP-1 in cell proliferation and survival. Oncogene 2001; 20: 2390–2400. 11402335 10.1038/sj.onc.1204383

[bib38] Zhang J, Zhang D, McQuade JS, Behbehani M, Tsien JZ, Xu M. c-fos regulates neuronal excitability and survival. Nat Genet 2002; 30: 416–420. 11925568 10.1038/ng859

[bib39] Kaufmann SH, Desnoyers S, Ottaviano Y, Davidson NE, Poirier GG. Specific proteolytic cleavage of poly(ADP-ribose) polymerase: an early marker of chemotherapy-induced apoptosis. Cancer Res 1993; 53: 3976–3985. 8358726

[bib40] Margolin N, Raybuck SA, Wilson KP, Chen W, Fox T, Gu Y et al. Substrate and inhibitor specificity of interleukin-1 beta-converting enzyme and related caspases. J Biol Chem 1997; 272: 7223–7228. 9054418 10.1074/jbc.272.11.7223

[bib41] Chaitanya GV, Steven AJ, Babu PP. PARP-1 cleavage fragments: signatures of cell-death proteases in neurodegeneration. Cell Commun Signal 2010; 8: 31. 21176168 10.1186/1478-811X-8-31 PMC3022541

[bib42] D'Amours D, Sallmann FR, Dixit VM, Poirier GG. Gain-of-function of poly(ADP-ribose) polymerase-1 upon cleavage by apoptotic proteases: implications for apoptosis. J Cell Sci 2001; 114: 3771–3778. 11707529 10.1242/jcs.114.20.3771

[bib43] Yamazaki H, Inoue T, Koizumi M, Yoshida K, Kagawa K, Shiomi H et al. Age as a prognostic factor for late local recurrence of early tongue cancer treated with brachytherapy. Anticancer Res 1997; 17: 4709–4712. 9494593

[bib44] Toualbi K, Guller MC, Mauriz JL, Labalette C, Buendia MA, Mauviel A et al. Physical and functional cooperation between AP-1 and beta-catenin for the regulation of TCF-dependent genes. Oncogene 2007; 26: 3492–3502. 17146436 10.1038/sj.onc.1210133

[bib45] Hwang SG, Yu SS, Lee SW, Chun JS. Wnt-3a regulates chondrocyte differentiation via c-Jun/AP-1 pathway. FEBS Lett 2005; 579: 4837–4842. 16099458 10.1016/j.febslet.2005.07.067

[bib46] Wang H, Zhou M, Brand J, Huang L. Inflammation and taste disorders: mechanisms in taste buds. Ann N Y Acad Sci 2009; 1170: 596–603. 19686199 10.1111/j.1749-6632.2009.04480.x PMC2729510

[bib47] Wang H, Zhou M, Brand J, Huang L. Inflammation activates the interferon signaling pathways in taste bud cells. J Neurosci 2007; 27: 10703–10713. 17913904 10.1523/JNEUROSCI.3102-07.2007 PMC2096741

[bib48] Li J, Chen K, Yan J, Wang Q, Zhao X, Yang X et al. Increased sucrose intake and corresponding c-Fos in amygdala and parabrachial nucleus of dietary obese rats. Neurosci Lett 2012; 525: 111–116. 22884642 10.1016/j.neulet.2012.07.053

[bib49] Hadamitzky M, Bosche K, Engler A, Schedlowski M, Engler H. Extinction of conditioned taste aversion is related to the aversion strength and associated with c-fos expression in the insular cortex. Neuroscience 2015; 303: 34–41. 26126924 10.1016/j.neuroscience.2015.06.040

[bib50] Lin JY, Roman C, Arthurs J, Reilly S. Taste neophobia and c-Fos expression in the rat brain. Brain Res 2012; 1448: 82–88. 22405689 10.1016/j.brainres.2012.02.013 PMC3313599

[bib51] Koh MT, Wilkins EE, Bernstein IL. Novel tastes elevate c-fos expression in the central amygdala and insular cortex: implication for taste aversion learning. Behav Neurosci 2003; 117: 1416–1422. 14674859 10.1037/0735-7044.117.6.1416

[bib52] Yasoshima Y, Sako N, Senba E, Yamamoto T. Acute suppression, but not chronic genetic deficiency, of c-fos gene expression impairs long-term memory in aversive taste learning. Proc Natl Acad Sci USA 2006; 103: 7106–7111. 16636292 10.1073/pnas.0600869103 PMC1459025

[bib53] Curtis BJ, Radek KA. Cholinergic regulation of keratinocyte innate immunity and permeability barrier integrity: new perspectives in epidermal immunity and disease. J Invest Dermatol 2012; 132: 28–42. 21918536 10.1038/jid.2011.264 PMC4648359

[bib54] Pumplin DW, Yu C, Smith DV. Light and dark cells of rat vallate taste buds are morphologically distinct cell types. J Comp Neurol 1997; 378: 389–410. 9034899 10.1002/(sici)1096-9861(19970217)378:3<389::aid-cne7>3.0.co;2-#

[bib55] Lawton DM, Furness DN, Lindemann B, Hackney CM. Localization of the glutamate-aspartate transporter, GLAST, in rat taste buds. Eur J Neurosci 2000; 12: 3163–3171. 10998100 10.1046/j.1460-9568.2000.00207.x

[bib56] Bartel DL, Sullivan SL, Lavoie EG, Sevigny J, Finger TE. Nucleoside triphosphate diphosphohydrolase-2 is the ecto-ATPase of type I cells in taste buds. J Comp Neurol 2006; 497: 1–12. 16680780 10.1002/cne.20954 PMC2212711

[bib57] Vandenbeuch A, Clapp TR, Kinnamon SC. Amiloride-sensitive channels in type I fungiform taste cells in mouse. BMC Neurosci 2008; 9: 1. 18171468 10.1186/1471-2202-9-1 PMC2235881

[bib58] Gao Y, Toska E, Denmon D, Roberts SG, Medler KF. WT1 regulates the development of the posterior taste field. Development 2014; 141: 2271–2278. 24803588 10.1242/dev.105676 PMC4034425

[bib59] Hacker K, Laskowski A, Feng L, Restrepo D, Medler K. Evidence for two populations of bitter responsive taste cells in mice. J Neurophysiol 2008; 99: 1503–1514. 18199819 10.1152/jn.00892.2007

[bib60] Maliphol AB, Garth DJ, Medler KF. Diet-induced obesity reduces the responsiveness of the peripheral taste receptor cells. PLoS One 2013; 8: e79403. 24236129 10.1371/journal.pone.0079403 PMC3827352

[bib61] Rebello MR, Maliphol AB, Medler KF. Ryanodine receptors selectively interact with L type calcium channels in mouse taste cells. PLoS One 2013; 8: e68174. 23826376 10.1371/journal.pone.0068174 PMC3694925

[bib62] Rebello MR, Medler KF. Ryanodine receptors selectively contribute to the formation of taste-evoked calcium signals in mouse taste cells. Eur J Neurosci 2010; 32: 1825–1835. 20955474 10.1111/j.1460-9568.2010.07463.x PMC2994989

[bib63] Szebenyi SA, Laskowski AI, Medler KF. Sodium/calcium exchangers selectively regulate calcium signaling in mouse taste receptor cells. J Neurophysiol 2010; 104: 529–538. 20463203 10.1152/jn.00118.2010 PMC2904227

[bib64] Laskowski AI, Medler KF. Sodium-calcium exchangers contribute to the regulation of cytosolic calcium levels in mouse taste cells. J Physiol 2009; 587: 4077–4089. 19581381 10.1113/jphysiol.2009.173567 PMC2756439

[bib65] Hacker K, Medler KF. Mitochondrial calcium buffering contributes to the maintenance of Basal calcium levels in mouse taste cells. J Neurophysiol 2008; 100: 2177–2191. 18684902 10.1152/jn.90534.2008 PMC2576209

[bib66] Tsompana M, Valiyaparambil S, Bard J, Marzullo B, Nowak N, Buck MJ. An automated method for efficient, accurate and reproducible construction of RNA-seq libraries. BMC Res Notes 2015; 8: 124. 25879881 10.1186/s13104-015-1089-9 PMC4391147

[bib67] Sapkota D, Chintala H, Wu F, Fliesler SJ, Hu Z, Mu X. Onecut1 and Onecut2 redundantly regulate early retinal cell fates during development. Proc Natl Acad Sci USA 2014; 111: E4086–E4095. 25228773 10.1073/pnas.1405354111 PMC4191802

[bib68] Starostik MR, Rebello MR, Cotter KA, Kulik A, Medler KF. Expression of GABAergic receptors in mouse taste receptor cells. PLoS One 2010; 5: e13639. 21049022 10.1371/journal.pone.0013639 PMC2964312

[bib69] Sevigny J, Sundberg C, Braun N, Guckelberger O, Csizmadia E, Qawi I et al. Differential catalytic properties and vascular topography of murine nucleoside triphosphate diphosphohydrolase 1 (NTPDase1) and NTPDase2 have implications for thromboregulation. Blood 2002; 99: 2801–2809. 11929769 10.1182/blood.v99.8.2801

